# Enhanced Biphasic Reactions in Amphiphilic Silica Mesopores

**DOI:** 10.1021/acs.jpcc.3c07477

**Published:** 2024-01-18

**Authors:** Guolin Zhao, Yao Li, Wen Zhen, Jie Gao, Yunjiao Gu, Bing Hong, Xia Han, Shuangliang Zhao, Marc Pera-Titus

**Affiliations:** † Eco-Efficient Products and Processes Laboratory (E2P2L), UMI 3464 CNRS − Solvay, 3966 Jin Du Road, Xin Zhuang Ind. Zone, Shanghai 201108, China; ‡ State Key Laboratory of Chemical Engineering, East China University of Science and Technology, Shanghai 200237, China; § School of Chemistry and Chemical Engineering, Guangxi University, Nanning 530004, China; ∥ Cardiff Catalysis Institute, School of Chemistry, Cardiff University, Main Building, Park Place, Cardiff CF10 3AT, U.K.

## Abstract

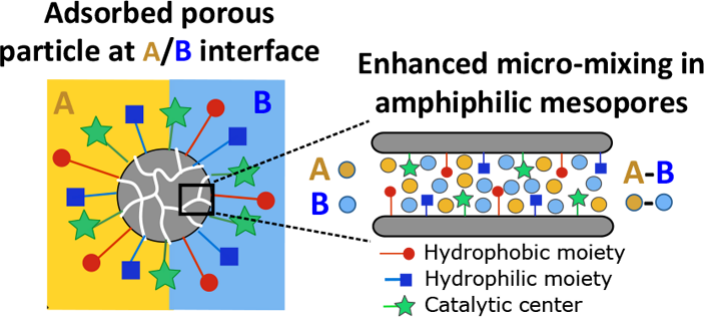

In this study, we investigated the effect of the pore volume and mesopore size of surface-active catalytic organosilicas on the genesis of particle-stabilized (Pickering) emulsions for the dodecanal/ethylene glycol system and their reactivity for the acid-catalyzed biphasic acetalization reaction. To this aim, we functionalized a series of fumed silica superparticles (size 100–300 nm) displaying an average mesopore size in the range of 11–14 nm and variable mesopore volume, with a similar surface density of octyl and propylsulfonic acid groups. The modified silica superparticles were characterized in detail using different techniques, including acid–base titration, thermogravimetric analysis, TEM, and dynamic light scattering. The pore volume of the particles impacts their self-assembly and coverage at the dodecanal/ethylene glycol (DA/EG) interface. This affects the stability and the average droplet size of emulsions and conditions of the available interfacial surface area for reaction. The maximum DA-EG productivity is observed for A200 super-SiNPs with a pore volume of 0.39 cm^3^·g^–1^ with an interfacial coverage by particles lower than 1 (i.e., submonolayer). Using dissipative particle dynamics and all-atom grand canonical Monte Carlo simulations, we unveil a stabilizing role of the pore volume of porous silica superparticles for generating emulsions and local micromixing of immiscible dodecanal and ethylene glycol, allowing fast and efficient solvent-free acetalization in the presence of Pickering emulsions. The micromixing level is interrelated to the adsorption energy of self-assembled particles at the DA/EG interface.

## 1 Introduction

Pickering emulsions are dispersions of two immiscible liquids stabilized by colloidal particles.^1−3^ They are extensively used in a variety of fields, including pharmaceutics, drug delivery, cosmetics, chemical synthesis, food industry, and oil recovery.^4,5^ Pickering emulsions can be employed as a platform for engineering multiphase microreactors comprising two immiscible reagents and a solid catalyst.^6,7^ These systems combine the advantages of homogeneous and heterogeneous catalysts, i.e., high activity and selectivity, easy phase separation, catalyst reuse, and compartmentalization of reactants and products.

Surface-active particles bearing catalytic centers have been developed to enhance the reactions at the liquid–liquid interface. In particular, organosilica particles (SiNPs) bearing sulfonic acid groups can behave as highly active catalysts for industrially relevant biphasic reactions, including acetalization, hydrolysis, etherification, esterification, transesterification, acylation, and Suzuki C–C coupling.^8−14^ The enhanced catalytic activity of these systems is typically attributed to the generation of large interfacial areas and short diffusion paths, promoting mass transfer between the phases and the surface of the catalytic particles.

The microenvironment around self-assembled particles in emulsions is known to affect the activity/selectivity of reactions by governing the coadsorption, segregation, and diffusion of reactants in the vicinity of catalytic active centers.^15,16^ The forces participating within the interparticle porosity can induce nanoscopic effects promoting the solubility between immiscible reactants.^17,18^ Also, the spatial assembly of catalytic particles at the inner/outer interfacial layer of emulsion droplets can affect the catalytic selectivity.^19^ Particle-free liquid–liquid interfaces can exhibit other nanoscopic phenomena, such as interfacial acidification/basification in water–oil systems,^20−23^ local solvation,^24,25^ surface charge,^26^ presence of diffusion barriers,^27^ and molecule^28−30^/catalyst^31−33^ orientation. Recent experiments and simulations have demonstrated the ability of high-surface electric fields (of the order of MV/cm) that can occur at the gas–water and water–oil interface due to preferential adsorption of HO^–^ species,^34−38^ to accelerate reactions.

**Figure 1:**
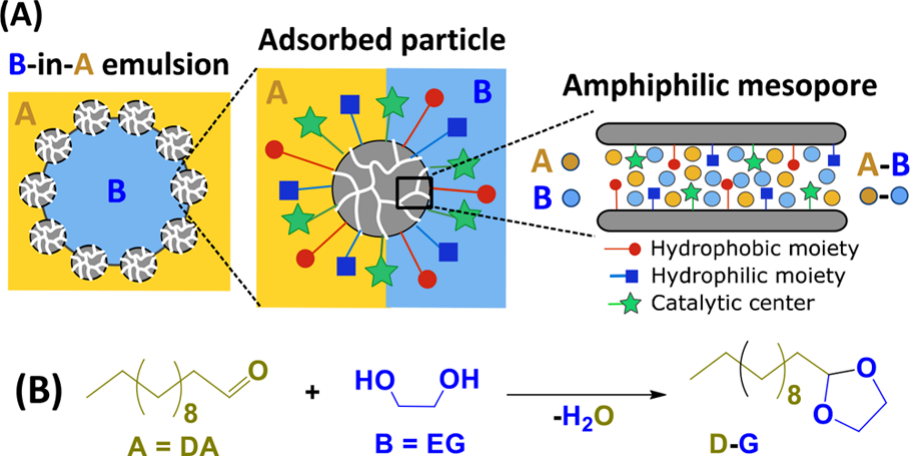
Pickering emulsions stabilized by silica super-SiNPs with amphiphilic mesopores and local intrapore micromixing for biphasic reactions between two immiscible reagents.

Herein, we unravel the key role of amphiphilic mesopores in simultaneously promoting particle adsorption at the liquid–liquid interface and local micromixing of the phases near the catalytic active centers, enhancing the rate of biphasic reactions (Figure 1A). To this aim, we combined emulsification and catalytic experiments using mesoporous organosilica with dissipative particle dynamics (DPD) and all-atom grand canonical Monte Carlo (GCMC) simulations. We selected the model acid-catalyzed acetalization reaction of dodecanal (DA) with ethylene glycol (EG) as an illustrative example of an oil/oil reaction commonly found upon biomass upgrading (Figure 1B).^8,39−44^

## 2 Experimental Procedures

### 2.1 Materials

The fumed silica particles with variable textural properties (Aerosil series, AX super-SiNPs, 3.7 SiOH/nm^2^) were supplied by Evonik. 3-Mercaptopropyl trimethoxysilane (97%), trimethoxy(octyl) silane (97%), and cyclohexane (analytical grade), all supplied by Macklin, were used as reagents for the postfunctionalization of silica particles. DA (98%, analytical grade) and EG (95%, 250 ppm BHT), also supplied by Macklin, were used for the emulsification tests and biphasic acetalization reactions. Absolute ethanol (analytical grade) and hydrochloric acid (37%, analytical grade) were purchased from Sinopharm Chemical Reagent Co Ltd. Hydrogen peroxide (30%, analytical grade) and Shanghai Wokai Biotechnology Co. Ltd. *p*-Toluenesulfonic acid monohydrate (PTSA, 98.5%) was supplied by TCI-EP and used as acid catalyst for oxidizing thiol groups on the AX super-SiNPs. Nitrogen (99.999%, Siling Gas) was used as an inert gas for the reactions. All the chemicals were used as received without further purification. Deionized water was used with a resistivity of 18.2 MΩ.

### 2.2 Functionalization of AX Super-SiNPs

The protocol for preparing the functionalized AX super-SiNPs was adapted from previous studies.^45^ Briefly, a given silica sample (2 g) was first dispersed in a water solution acidified with 5% HCl (200 mL) at room temperature for 4 h under stirring. Then, the sample was centrifuged, and the as-obtained solid was washed with deionized water at least three times until neutral pH and dried in an oven overnight at 90 °C.

After activation, the sample was subjected to functionalization. The sample (500 mg) was added to a glass flask and was pretreated at 110 °C under vacuum for 12 h. After cooling down to room temperature, cyclohexane (50 mL) with 3-mercaptopropyl trimethoxysilane (0.5 mL), trimethoxy(octyl)silane (2.8 mL), and PTSA (3 mg) was added under N_2_ atmosphere and mild stirring. The suspension was first heated at 40 °C for 1 h to dissolve the silane precursors and was further heated to 80 °C for 4 h. After this period, the suspension was cooled down to room temperature, centrifuged, and washed three times with cyclohexane and ethanol to remove the unreacted residues and the solvent, and the final solid was dried in an oven overnight at 90 °C. The −SH groups were further oxidized at 40 °C for 24 h using an aqueous solution of H_2_O_2_ (30%) and ethanol (volume ratio = 1:1) and a silica-to-solvent weight ratio of 1:60.

### 2.3 Characterization of AX Super-SiNPs

The acidity of AX super-SiNPs was measured by acid–base titration using a pH meter. The samples (50 mg) were first suspended in 5 mL of an aqueous solution of NaCl (1 M) and ethanol (5 mL) for 24 h. The acidity of the solution was titrated using an aqueous solution of NaOH (0.01 M).

Thermogravimetric analysis (TGA) was used for measuring the grafting degree of the octyl and propylsulfonic acid groups on the AX super-SiNPs. The measurements were performed on a TA SDT Q600 apparatus. The samples (∼10 mg in a 100 μL alumina crucible with an alumina lid) were heated from room temperature to 1000 °C using a heating rate of 10 °C.min^–1^ under air atmosphere at a flow rate of 100 mL (STP)·min^–1^.

The textural properties of the particles were measured by N_2_ adsorption at −196 °C using a Micromeritics ASAP 2010 Surface Area Analyzer. The surface areas were calculated by the Brunauer–Emmett–Teller (BET) method in the relative pressure range 0.05 < *P*/*P*_0_ < 0.30, while the pore volume was measured at *P*/*P*_0_ = 0.97. The average pore size was measured using the Barrer–Joyner–Halenda (BJH) method. Prior to the measurements, the catalysts were degassed at 150 °C for 3 h to remove the adsorbed moisture.

The particle size and distribution of AX super-SiNPs were determined by high-resolution transmission electron microscopy (HR-TEM) on a JEOL-JEM 1400 microscope from TA Instruments with an accelerating voltage of 200 kV. The images were analyzed by ImageJ software. Besides, the particle size distribution of the solvated particles was measured by dynamic light scattering (DLS) in a Malvern Nano-ZS instrument using light scattering cells of 10 mm.

### 2.4 Emulsification Tests

The EG-in-DA emulsions were prepared as follows. First, EG (1.67 mL) and a variable weight of the given AX super-SiNP were added to a 10 mL test tube. After 10 min of ultrasonication, DA (3.33 mL) previously melted at 70 °C was added (2:1 EG/DA molar ratio), and the final system was heated to 50 °C for 15 min. Then, the system was homogenized using an ultraturrax at 13,000 rpm for 15 min, and the emulsified system was transferred to a thermostated bath (Julabo) at 50 °C for monitoring the stability.

Assuming spherical droplets, the interfacial area of the emulsions, *S*_E,int_, can be measured from the volume of the dispersed phase, *V*_E_, and the average diameter of the emulsion droplets, D_E_, using the expression
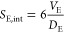
1The interfacial coverage of particles was computed by assuming full adsorption of particles using the expression^3^
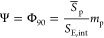
2where *S̅*_p_ is the effective surface area of adsorbed particles at the DA/EG interface by assuming a contact angle of 90*°*, which accounts for the cross-section of the particles in contact with the droplet surface, and *m*_p_ is the weight of particles.

*S̅*_p_ can be computed from the specific surface area of the super-SiNPs (cross-section) using the expression
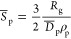
3which includes explicitly the surface roughness of the particles, *R*_g_ (−).

The interfacial density of particles, Γ_p_, was calculated by dividing the total number of particles, *N*_p_, by the interfacial area, *S*_E,int_. The following expression was used:
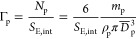
4The emulsion volume was measured by direct inspection of EG-in-DA emulsions using a Nikkon D300s camera equipped with a macro lens (AF-S Macro Nikkon 105 mm 1:2.8G ED) and using NKremote software. The continuous phase was assessed by dropping one droplet of the emulsion into DA and EG separately. The droplet size distribution was measured using an Olympus IX-51 light transmission microscope equipped with 10× ocular, 4×, 10×, 40×, and 100× objectives, and DP2-BSM software. Visilog software was used to measure the individual droplet size. The average droplet size was measured as an arithmetic average of the individual droplet sizes.

The EG/DA interfacial tension was measured on a Sigma 700 tensiometer (Biolin Scientific AB) equipped with a Wilhelmy plate and a Du Noüy ring set at 3 mm/min for low interfacial tensions.

### 2.5 Catalytic Tests

The model acetalization reaction between DA and EG toward the cyclic acetal product (DA-EG) was used for qualifying the catalytic activity of the AX super-SiNPs. The catalytic tests were conducted in both the presence and absence of emulsion. In the former case, EG (1.0 mL) and the given AX super-SiNP (30 mg) were added to a 10 mL test tube. After 10 min of ultrasonication, DA (2.0 mL), previously melted at 70 °C, was added to achieve a 1:2 DA/EG molar ratio, and the final system was heated to 50 °C for 15 min. The system was further homogenized using an ultraturrax at 13,000 rpm for 15 min. The emulsified system was then transferred to a thermostated bath (Julabo), and the reaction was conducted at 60 °C for 1 h under mild stirring.

The catalytic tests in the absence of emulsion were carried out as follows. A given AX super-SiNP (0.3 g) was added to a glass tube. Equimolar amount of EG and DA were impregnated into the pore volume using a volume excess of 20–50% compared to the pore volume, and the impregnated particles were submitted to reaction at 60 °C for 1 h. Three impregnation methods were implemented:

Method i: First impregnation of EG, followed by DA.
Method ii: First impregnation of DA, followed by EG.
Method iii: Coimpregnation of EG and DA.

After the reaction, isopropanol (5 mL) was added to generate a sole phase, the final solution was centrifuged at 8,000 rpm for 10 min, the supernatant solution was recovered using a syringe, and 1,2-dichlorobenzene (0.1 g) was introduced as an internal standard. The solution was analyzed using an Agilent 7890 GC equipped with a FID detector and a HT-5 capillary column (length 30 m, i.d. 0.32 mm, film thickness 0.10 μm). Mass balance errors were controlled within 5% for all of the catalytic tests. The yield of the product (DA-EG) was defined as the mole number of DA-EG produced divided by the initial mole number of the limiting reactant (EG). The error of the DA-EG yield as quantified by GC was in the range of 5–10%. The catalyst productivity (*P*) was defined as the mole number DA-EG per mole of H^+^ after 30 min of reaction. To identify and quantify the formation of the hemiacetal intermediate, additional analyses were conducted by nuclear magnetic resonance (^1^H NMR) on a Bruker Avance III 300 spectrometer operating at 600 MHz resonance frequency using CDCl_3_ and tetramethylsilane as a reference.

The DA conversion (DA = limiting reactant) and DA-EG yield were calculated by interpolation of the corresponding calibration curves using biphenyl as internal standard as follows:
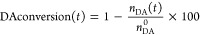
5
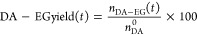
6where *n*_DA_^0^ and *n*_DA_(*t*) refer to the mole number of DA at time = 0 and time = *t*, respectively, and *n*_DA-EG_(*t*) is the mole number of the acetalization product (DA-EG) at time = *t*.

## 3 Simulation Details

### 3.1 Dissipative Particle Dynamics

#### 3.1.1 Theoretical Basis

DPD is a coarse-grained simulation method that preserves the essential properties of matter while simplifying the freedom of the system. Therefore, research on large-scale systems has a better applicability than other simulation methods. In DPD simulations, the bead motion is described by Newton’s equation of motion. The external force (**F**_*i*_) of each bead is composed of three components: conservative force (**F**_ij_^C^), dissipative force (**F**_ij_^D^), and random force (**F**_ij_^R^).^46−48^
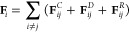
7The three different component forces represent different contributions to the system. First, the conservative force (**F**_ij_^C^) is expressed by the contribution of the soft interaction force and is also the primary force over the bead. This force can be accounted for by the expression
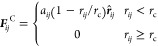
8where *r*_*ij*_ = *r*_*i*_*– r*_*j*_ is the centroid distance between the *i*th and *j*th beads, **r̂**_*ij*_ = *r*_*ij*_/|*r*_*ij*_| is the unit vector of the direction from the *i*th to the *j*th bead, *r*_C_ is the cutoff radius of the pairwise bead interaction, setting the basic length-scale in DPD simulations, and the coefficient *a*_*ij*_ represents the maximum repulsion between two interacting beads. When *i* equals *j*, the interaction parameter of *a*_*ii*_ can be expressed as follows^49^:
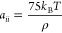
9where ρ is the bead number density, *k*_B_ is the Boltzmann constant, and *T* is the absolute temperature. *k*_B_*T* represents the reduced energy in DPD simulations and is set to 1. When the bead number density of the system is 3, the intraspecies interactions (*a*_*ii*_) is 25. In parallel, the interspecies interaction parameters (*a*_*ij*_) can be calculated from the Flory–Huggins binary interaction parameters (χ_*ij*_) as a function of ρ^50^
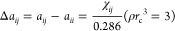
10
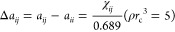
11The Flory–Huggins parameters can be computed from the Hansen solubility parameters^3^ using the expressions
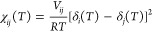
12
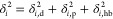
13where δ_*i*_ is the Hansen solubility parameter, δ_*i*,d_, δ_*i*,p_, and δ_*i*,hb_ represent the dispersion, polar, and hydrogen-bonding components, *V*_*ij*_ is the partial molar volume of the DPD bead, and *R* is the universal constant of perfect gases.

In addition to the conservative force, the dissipative force (**F**_*ij*_^D^) and the random force (**F**_*ii*_^R^) represent the viscosity contribution and the thermal fluctuation contribution of the system, respectively. These forces can be expressed as follows:
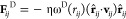
14
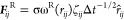
15where η is the friction coefficient, *v*_*ij*_*= v*_*i*_*– v*_*j*_ is the relative velocity between the *i*th and *j*th beads, **σ** is the amplitude of noise, ξ_*ij*_ is a random number with a zero mean and unit variance with the Gaussian distribution, Δ*t* is the time step, and *w*^D^(*r*_*ij*_) and *w*^R^(*r*_*ij*_) are the weight functions for dissipative and random forces, respectively, which are related by the expression
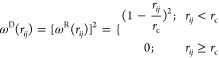
16
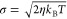
17Along this work, we used reduced DPD units to boost the calculations. In particular, we used *r*_c_ and *k*_B_*T* as length and energy units, respectively, with nominal values of 7.11 and 1.0. The mass of beads, *m*, was set equal to 1.

#### 3.1.2 Models and Interaction Parameters

Coarse-grained DPD was used to compute the interfacial contact angles of the simulated super-SiNPs at variable pore volumes and surface properties. EG was modeled as a single bead (EG), while DA comprised the assembly of three propane beads (Pe) and one propanal bead (PA) (see SI, Figure S1A,B). The simulated super-SiNP, with a radius of *R* = 3.022 nm (4.25*r*_c_), consisted of an assembly of primary SiNPs (*n* = 6–13) with *r* = 0.889 nm (1.25*r*_c_), while the distance between primary SiNPs was 3*r*_c_ (see SI, Figures S1C and S2). The surface of the SiNPs was covered by a layer of hydrophilic (HL) and hydrophobic (HB) beads with a total density of 3.5 groups/nm^2^, which is compatible with the surface density of the experimental AX super-SiNPs. The HL beads were simulated using water (H_2_O) or hydroxyl (−OH) parameters, while propane (Pe) parameters were used for the HB beads. The interaction parameters, *a*_*ij*_, between the particle and reagent beads are listed in Table S1 (see SI). The pore volume of the simulated super-SiNPs evolved from 0.92 to 2.5 cm^3^·g^–1^ for super-SiNPs composed of 13 and 6 primary SiNPs, respectively, with a comparable pore size around 0.355 nm.

#### 3.1.3 Computational Details

The DPD simulations were performed by using the LAMMPS package. The simulation box size was set at *L*_*x*_ × *L*_*y*_ × *L*_*z*_ ≡ 25 × 15 × 15 *r*_c_^3^, where *L*_*i*_ refers to the length of the simulation box along the *i* direction. Orthorhombic boxes were used, and periodic boundary conditions were applied in all three directions. The *x*-direction was perpendicular to the DA/EG interface, which was considered planar. The reduced number density of the simulation cell was set to ρ = 3. The constants for the dissipative and random forces were set at η = 4.5 and σ = 3 (eqs 14 and 15), respectively, to keep the temperature constant at a scaled value of *k*_B_*T* = 1. The EG/DA volume ratio was 1:1 with EG and DA densities of 1.11 and 0.83 g/cm^3^, respectively. The simulation runs were established at 200,000 steps with a time step of 0.05 to ensure steady-state equilibrium.

#### 3.1.4 Calculation of Interfacial Contact Angles

The DA/EG interfacial contact angle, θ_DA/EG,C_, of the different super-SiNPs at the DA/EG interface was computed using the following expression derived geometrically from Figure S3 (see SI):
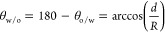
18where *d* is the distance between the nanoparticle center and the DA/EG interface, and *R* is the radius of the super-SiNPs. The interface was defined when the number density of DA or EG beads decreased from 90% to 10%.

#### 3.1.5 Calculation of the Dimensionless Adsorption Energy of Super-SiNPs

The dimensionless energy of adsorption of a single particle at the DA/EG interface, *E*_p,dim_, was computed using the interfacial contact angle using the expression
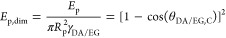
19In our approach, we considered the range 0.25–0.83 for *E*_p,dim_, corresponding to the θ_EG-DA,C_ range of 60–85° (DA/EG emulsions) and 95–120° (EG/DA emulsions) ensuring emulsion stability.

#### 3.1.6 Calculation of Theoretical DA/EG Ratios in the Pore Volume of Super-SiNPs

The theoretical DA/EG values in the pore volume of super-SiNPs [(DA/EG)_theo_] was estimated by taking into account homogeneous pore filling by EG and DA from each phase for the particles adsorbed at the DA/EG interface using the following expression:
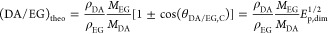
20

### 3.2 Grand Canonical Monte Carlo

The all-atom GCMC simulations of DA and EG coadsorption in silica slits were carried out using the adsorption module (Sorption) in Materials Studio. In the GCMC simulations, the pressure of the system was kept constant, that is, *P*_EG_ + *P*_DA_ = 100 kPa. To have a flexible structure in the simulations, we selected the configuration bias option in the calculation method and the COMPASS force field. The size of the slits was set to 10 × 4 × *Z* nm^3^, with *Z* = 2, 3, 4, 5 nm (slit size). The total number of simulation steps was 500,000. The first 250,000 steps were used to reach equilibrium, while the remaining 250,000 steps were used for data generation and analysis. The temperature was set to 298 K.

## 4 Results and Discussion

### 4.1 Synthesis of AX Super-SiNPs

A series of commercial fumed silicas (Aerosil) was acquired, consisting of an assembly of primary SiNPs defining superparticles (i.e., super-SiNPs) with comparable size but variable pore volume (Figure S4). The super-SiNPs were functionalized with propylsulfonic acid (C_3_SO_3_H) and octyl (C_8_) groups using a protocol consisting of 3 steps (see SI)^45^: (i) activation in a 5 wt % HCl/water solution at room temperature for 4 h, (ii) grafting with (3-mercaptopropyl) trimethoxysilane and trimethoxy(octyl)silane precursors in cyclohexane with a 1:4 molar ratio using p-toluenesulfonic acid as catalyst at 80 °C for 4 h under N_2_ atmosphere, and (iii) oxidation of thiol (−SH) groups into sulfonic acid groups using H_2_O_2_ (30 wt %) in water/ethanol (1:1 v/v) at 40 °C for 24 h. Six samples were prepared, hereinafter referred to as AX (X = 50–300). The samples show a comparable average size of about 112–184 nm (DLS), a specific surface area (*S*_BET_) and pore volume (*V*_p_) evolving from 39 to 303 m^2^ g^–1^ and from 0.076 to 0.56 cm^3^ g^–1^, respectively, and a comparable average pore size of about 11–14 nm (Table 1, columns 2–5). The surface density of C_3_SO_3_H and C_8_ groups fell into the range 0.16–0.97 and 0.36–1.6 groups/nm^2^, respectively, whereas the density of SiOH groups was about 0.7–1.5 groups/nm^2^ for all samples as inferred from combined acidity and TGA measurements (Table 1, columns 6–7).

**Table 1:** Main Properties of Functionalized AX Super-SiNPs

AX	*D*_part_ (nm)^a^	*S*_BET_ (m^2^/g)^b^	*V*_p_ (cm^3^/g)^b^	*d*_pore_ (nm)^b^	C_3_SO_3_H (mmol/g)^c^	C_8_/C_3_SO_3_H (mol:mol)^d^
A380	160 (20)	303 (−)	0.55	10.2	0.09 (0.16)	2.22 (0.36)
A300	141 (15)	272 (16)	0.52	11.0	0.20 (0.62)	1.93 (1.2)
A200	153 (18)	194 (9.2)	0.39	11.2	0.12 (0.42)	2.31 (0.98)
A150	184 (25)	100 (−)	0.22	11.9	0.14 (0.65)	0.78 (0.50)
A90	199 (38)	84 (12)	0.14	10.9	0.11 (0.88)	1.83 (1.6)
A50	180 (75)	38 (7.2)	0.076	13.8	0.06 (0.97)	1.70 (1.6)

a Average particle diameter measured by DLS; in brackets, average particle diameters measured by TEM.

b Measured from N_2_ adsorption at −196 °C after grafting; in brackets, micropore surface areas measured by the *t*-plot method.

c Measured by acid–base titration after NaCl solution exchange; in brackets, C_3_SO_3_H number per nm^2^.

d Measured from the acidity and TGA in the range 200–400 °C; in brackets, C_8_ number per nm^2^.

### 4.2 DA/EG Emulsification and Catalytic Properties of AX Super-SiNPs

**Figure 2:**
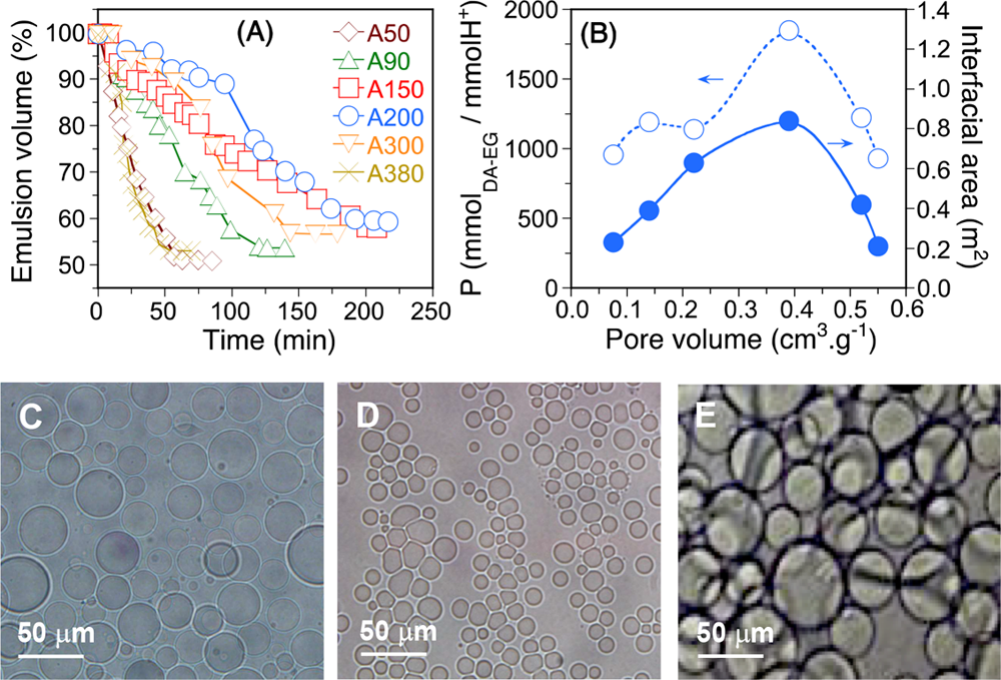
(A) Time-evolution of EG-in-DA emulsions generated by super-SiNPs; (B) evolution of the productivity and interfacial area after emulsion stabilization; and (C–E) optical images of emulsion droplets generated with A50, A200, and A380 super-SiNPs after 5 min. Emulsification conditions: DA/EG ratio = 2:1 (v/v), 1.1 wt % SiNP (50 mg), 50 °C, homogenization at 13,000 rpm for 5 min. Reaction conditions: DA/EG ratio = 2:1 (v/v), 0.7 wt % SiNP (30 mg), 60 °C, 1 h, homogenization at 13,000 rpm for 5 min.

We investigated the emulsification properties of AX super-SiNPs for the DA/EG system (Table S2). The emulsification tests were conducted at a 1:2 EG/DA volume ratio and 50 °C using 1.1 wt % SiNPs by dispersing first the SiNPs in the EG phase and then homogenizing the mixture using an ultraturrax at 13,000 rpm for 15 min. In all cases, EG-in-DA emulsions are formed. The emulsion stability increases with the pore volume of the AX super-SiNPs in the range from 0.076 (A50) to 0.39 cm^3^·g^–1^ (A200), with an emulsion volume reaching 60% after 3 h (Figure 2A). Above 0.39 cm^3^·g^–1^, super-SiNPs A300 and A380 exhibit much lower emulsion stability, with only 50% emulsion volume after 1 h. In line with these observations, the average droplet diameter decreases with the pore volume of AX super-SiNPs in the range from 0.076 to 0.39 cm^3^·g^–1^ and increases further for particles A300 and A380 displaying larger pore volumes (Figure S5). This trend results in a volcano plot of the interfacial area of the emulsions (*S*_E,int_) as a function of the pore volume of super-SiNPs with a maximum value of 0.39 cm^3^·g^–1^ (A200) (Figure 2B, right). Representative optical micrographs of the emulsions generated by A50, A200, and A30 are depicted in Figure 2C–E.

The above trends in the emulsion stability and average droplet size can be rationalized by the different coverage of super-SiNPs (Ψ = Φ_90_) at the DA/EG interface, computed from eq 2 for a theoretical interfacial contact angle of 90°, as a function of the pore volume of the super-SiNPs. Two dissimilar patterns are observed (Figure S6). On the one hand, for a pore volume lower than 0.39 cm^3^·g^–1^ (A200), the interfacial coverage by super-SiNPs is lower than 1 (Ψ ∼ 0.44–0.95) with an interfacial density of particles in the range of 17–37 SiNP/μm^2^. On the other hand, for higher pore volumes, the interfacial coverage is higher than 1 (Ψ ∼ 1.24–1.69) with an interfacial density of particles higher than 80 SiNP/μm^2^. This different self-assembly pattern can be explained by competitive particle–particle interactions compared with particle–interface interactions for super-SiNPs with pore volumes higher than 0.39 cm^3^·g^–1^ that might favor multilayer particle adsorption at the DA/EG interface with and exceed the critical mass fraction (CMF) of particles.^51^ In all cases, no super-SiNPs are observed in suspension after emulsification, pointing to their complete adsorption at the DA/EG interface.

Given the variable interfacial coverage of super-SiNPs as a function of their pore volume, we investigated their catalytic properties in the acetalization reaction of DA with EG at 60 °C for 1 h and 0.7 wt % particle loading. The DA-EG productivity displays a volcano plot with the pore volume of the super-SiNPs that correlates well with the trend observed for the interfacial area of the emulsions (Figure 2B, left), with a maximum value of about 2000 mmol DA-EG/mmolH^+^ for a pore volume of 0.39 cm^3^·g^–1^ (A200).

Overall, this body of results clearly shows that the pore volume of super-SiNPs impacts their self-assembly and, accordingly, their coverage at the DA/EG interface. This affects the stability and the average droplet size of EG-in-DA emulsions and conditions, in turn, the available interfacial surface area for reaction. The maximum DA-EG productivity is observed for A200 super-SiNPs with a pore volume of 0.39 cm^3^·g^–1^ with an interfacial coverage by particles lower than 1 (i.e., submonolayer). This peculiar behavior prompted us to rationalize the effect of the pore volume on the emulsification properties of super-SiNPs.

### 4.3 Prediction of DA/EG Emulsification Properties of Super-SiNPs Using DPD

**Figure 3:**
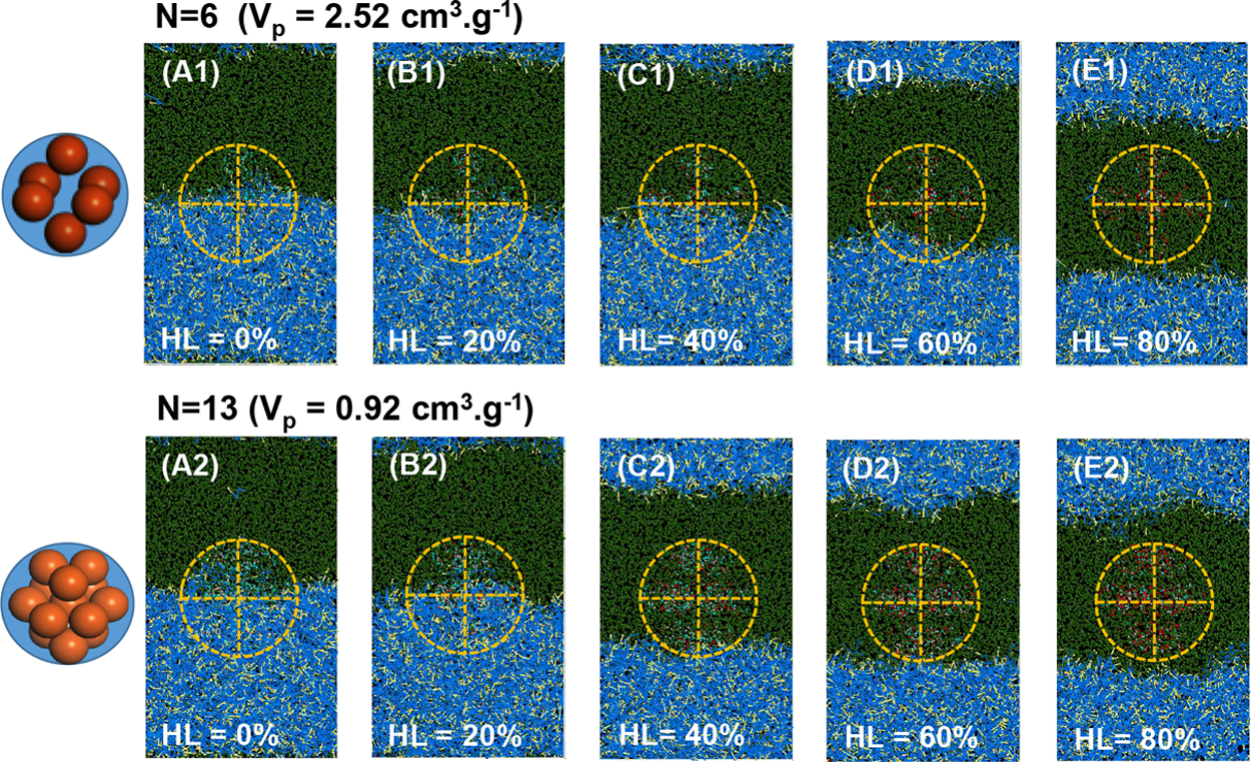
Snapshots showing the position of super-SiNPs in the DA/EG system: (A1–E1) 8NPs; and (A2–E2) 12NPs. Notation for A1–E2: DA (blue beads), EG (green beads), perimeter of super-SiNPs (yellow dotted line).

Coarse-grained DPD was used to compute the interfacial contact angles of simulated super-SiNPs at variable pore volumes and surface properties, as detailed in the Experimental section. We first evaluated the stability of the different super-SiNPs at the DA/EG interface by computing the interfacial contact angles and adsorption energies. Figure 3 shows the representative snapshots for super-SiNPs with the largest and lowest pore volumes, i.e., 6NPs [Figure 3(A1–E1)] and 13NPs [Figure 3(sA2–E2)]. A complete series of snapshots for all of the super-SiNPs is provided in Figures S7–S9 (see SI). Super-SiNPs with HL <60% can effectively adsorb at the DA/EG interface, while at HL >60%, super-SiNPs are mainly dispersed in the EG phase. At comparable hydrophilicity (i.e., HL = 20, 40, and 60%), super-SiNP adsorption and thus emulsion stability are affected by the pore volume. Therefore, as the hydrophilicity of small-SiNPs gradually increases, the stability of the super-SiNPs at the DA/EG interface gradually decreases.

**Figure 4:**
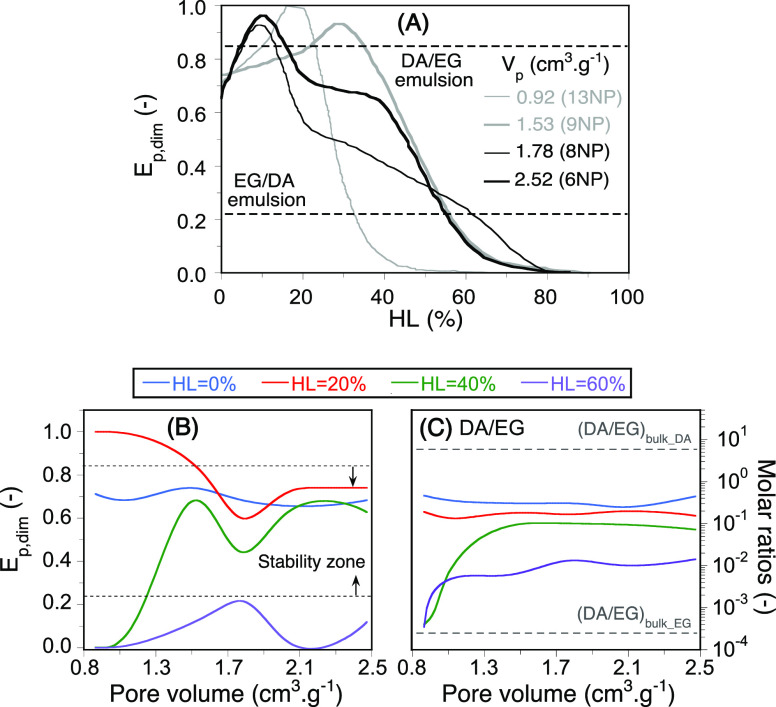
(A) Dimensionless adsorption energy (*E*_p,dim_) of super-SiNPs as a function of the hydrophilicity (HL) for different pore volumes; (B) *E*_p,dim_ of super-SiNPs at the DA/EG interface at variable pore volume and hydrophilicity; and (C) DA/EG molar ratios within the pore volume of super-SiNPs.

From the calculations, we computed the dimensionless interfacial adsorption energy [*E*_p,dim_ = (1 – cos θ)^2^] of a single super-SiNP as a function of the pore volume at different HL values using eq 19 (Figure 4A, B). The stability zone for emulsification can be established in the range of 0.25 < *E*_p,dim_ < 0.83 (see SI for details).^52^*E*_p,dim_ shows a maximum for super-SiNPs with intermediate values (i.e., *n* = 6,7) (Figure 4A). At HL = 0%, *E*_p,dim_ lies within the stability zone irrespective of the pore volume, pointing out the formation of stable emulsions (Figure 4B). In contrast, at HL = 20%, *E*_p,dim_ decreases monotonously with the pore volume within the stability zone for values higher than 1.3 cm^3^·g^–1^. Increasing the hydrophilicity to HL = 30–40% decreases particle stability. Indeed, at lower pore volume (i.e., *n* = 13NP-8NP), super-SiNPs can hardly adsorb at the DA/EG interface. Higher pore volumes promote interfacial particle adsorption, especially for super-SiNPs with HL >20%. Noticeably, these theoretical results qualitatively capture the observed dependence of the emulsion stability and interfacial area of emulsion droplets (*S*_EG,int_) on the pore volume of the experimental AX super-SiNPs, and preferential formation of DA/EG emulsions at low HL (Figure 2A, B). Finally, regardless of the pore volume, hydrophilic super-SiNPs (HL > 60%) display poor stability, matching also the experimental observation.

### 4.4 EG/DA Micromixing in the Pore Volume of Super-SiNPs Computed by DPD

We further computed the microscopic distribution of EG and DA within the confined pore volume of the super-SiNPs (see SI, Table S3). Regardless of the pore volume, the EG concentration increases gradually in the amphiphilic pore space with the surface hydrophilicity of small-SiNPs, while the DA concentration decreases accordingly. This result leads to an increase in the average DA/EG molar ratio in the pore space (Figure 4C, Table S4), especially at HL = 0–40%, displaying the best emulsification properties. These ratios are larger than the theoretical values measured taking into account homogeneous pore filling by EG and DA from each phase for super-SiNPs adsorbed at the DA/EG interface according to eq 20 (see SI, Figure S10), pointing out a high degree of micromixing. The DA/EG ratios in the pore space are comprised within the bulk ratios in the EG and DA phases, i.e., (DA/EG)_bulk_EG_ = 2.5 × 10^–4^ and (DA/EG)_bulk_DA_ = 6.25, respectively, with a value in the range of 0.095–0.31 for HL = 0–40% and a pore volume range of 1.53–2.52 cm^3^·g^–1^. This observation points out that within the confined pore space, EG and DA exhibit better micromixing compared to the bulk phases, which provides a basis for enhanced catalytic activity compared with the bulk phase.

### 4.5 EG/DA Micromixing in Amphiphilic Mesopores Computed by All-Atom GCMC

**Figure 5:**
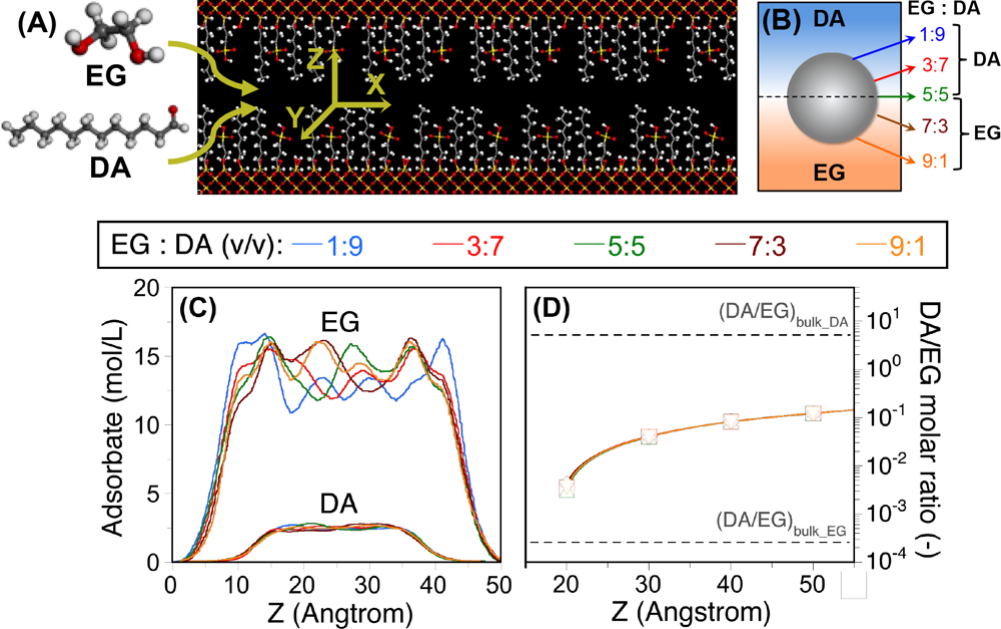
(A) Snapshot of an amphiphilic silica slit grafted with C_3_SO_3_H, C_8_, and SiOH groups with a surface density of 0.40, 1.0, and 0.6 groups/nm^2^, respectively; (B1–B2) concentration profile in a 5 nm slit simulated by all-atom GCMC at variable EG:DA bulk volume ratios; (C) scheme of super-SiNP adsorbed at the DA/EG interface showing different EG:DA bulk volume ratios; and (D) DA/EG molar ratios in the slit as a function of the width.

The microscopic distribution of DA and EG inside the pores in super-SiNPs impacts the DA/EG micromixing. On the basis of the coarse-grained DPD simulation study, we studied the microscopic distribution of EG and DA in slits of different widths through all-atom GCMC simulations for simultaneous coadsorption of EG and DA using the pore model depicted in Figure 5A (see SI and Figure S11 for more details). The slits (pore widths of 2, 3, 4, and 5 nm) were modified with C_3_SO_3_H, C_8_, and SiOH groups with a surface density of 0.40, 1.0, and 0.6 groups/nm^2^, respectively, that is compatible with the experimental values on AX super-SiNPs (Table 1). In the simulations, we considered variable EG:DA bulk volume ratios for adsorbed super-SiNPs at the DA/EG interface as a function of their distance from the interface. In this view, we selected five different EG:DA bulk volume ratios corresponding to different particle locations depicted in Figure 5B, that is 1:9, 3:7, 5:5, 7:3, and 9:1.

The concentration profile of DA and EG was simulated in slits with different widths in the range of 2–5 nm (Figures 5C and S12). EG gradually distributes within the slit, whereas DA is mainly located at the center of the slits for widths above 3 nm. However, DA can hardly enter 2 nm slits due to size exclusion. From these profiles, we computed the DA/EG molar ratios in the slits (Figure 5D, Tables S5–S6). No remarkable differences are observed using different EG:DA bulk volume ratios corresponding to different particle locations. However, the DA/EG molar ratio increases with the slit size with a value of about 0.12 for 5 nm slits. Remarkably, the DA/EG molar ratios computed by all-atom GCMC on pore slits are consistent with those computed by DPD for the entire pore volume. Overall, these results point out that both EG and DA can penetrate the pore volume of the experimental AX super-SiNPs when adsorbed at the DA/EG interface, with a mesopore size higher than 10 nm and provide higher DA/EG miscibility compared with the bulk DA and EG phases.

With these results in hand, we contrasted the predicted micromixing effects in amphiphilic mesopores with a series of catalytic activity tests on AX super-SiNPs listed in Table 1 but without emulsion. In these tests, DA and EG were first impregnated in the pore volume of the AX silica-SiNPs, followed by a reaction at 60 °C for 1 h (see the SI for details). Three impregnation methods were used: (i) EG was first impregnated, followed by DA; (ii) DA was first impregnated, followed by EG; and (iii) coimpregnation of EG and DA from a biphasic mixture. The results show a very high DA-EG yield (about 90%) for all super-SiNPs for methods (i) and (iii) (see for experimental details SI, Table S7). In contrast, method (ii) results in lower yields, suggesting a hindered diffusion of EG within DA-impregnated pores despite their large size (>10 nm). We also conducted two catalytic tests on nonfunctionalized A200 and A380 super-SiNPs (HL = 100%). In both cases, the DA-EG yield is low (13%) for impregnation methods (i) and (iii), and it is even lower (5%) for method (ii). Further, all-atom GCMC calculations on ungrafted slits, i.e., only including SiOH groups (3.5 groups/nm^2^) (not shown), display preferential EG adsorption and strong DA hindering, inhibiting the reaction, which is in line with the experimental observation.

## 5 Conclusions

In summary, the mesopore size and pore volume of surface-active (amphiphilic) catalytic silica superparticles were found to condition to an important extent their interfacial self-assembly and coverage for the EG/DA system and thus affect the stability and available interfacial surface area for the acetalization reaction between both reagents. The maximum DA-EG productivity is observed for A200 super-SiNPs with an average mesopore size of 11 nm and pore volume of 0.39 cm^3^·g^–1^, achieving a submonolayer interfacial coverage by particles. By contrasting the experimental emulsification results with DPD and all-atom GCMC simulations, we unveil a stabilizing role of the pore volume of porous silica superparticles for generating emulsions and local micromixing of immiscible DA and EG. The micromixing level correlates with the adsorption energy of superparticles at the liquid–liquid interface promoting emulsification. The results presented in this study open an avenue for the in silico design of surface-active mesoporous catalysts for industrial multiphase reactions in emulsion, with enhanced intrapore micromixing using the interfacial adsorption energy of particles as a descriptor.

## Supporting Information Available

Coarse-grained models for DPD simulations; structure of simulated super-SiNPs constituted by small primary particles with variable pore volume; example of super-SiNP adsorbed at the DA/EG interface and calculation of interfacial contact angles; HR-TEM micrographs of Aerosil particles; evolution of the average size of droplets as function of the average particle diameter of funcionalized super-SiNPs; evolution of the super-SiNP density at the DA/EG interface as a function of the pore volume of super-SiNPs; snapshots showing the position of super-SiNPs based on different primary NPs in the EG/DA system; evolution of DA/EG ratios in the pore volume of super-SiNPs measured from DPD simulations against theoretical values estimated taking into account homogeneous pore filling by EG and DA from each phase at the DA/EG interface; pore slits with sizes in the range 2–5 nm functionalized with C_3_SO_3_H, C_8_ and SiOH groups for all-atom GCMC simulations; EG and DA concentration profiles in slits at variable EG:DA bulk volume ratios; interaction parameters used in the DPD simulations; physicochemical properties of emulsions stabilized by AX super-SiNPs; concentration of EG and DA in the pore volume of super-SiNPs computed by DPD; average DA/EG ratios in the pore volume of super-SiNPs computed by DPD; concentration of EG and DA in slits of different sizes computed by all-atom GCMC; average DA/EG ratios in slits of different sizes computed by all-atom GCMC; and DA-EG yield in the acetalization reaction of DA and EG in impregnated AX super-SiNPs using methods i–iii^a^ (PDF)
