# Supplementary material for: Enhanced Biphasic Reactions in Amphiphilic Silica Mesopores
Source: J Phys Chem C Nanomater Interfaces. 2024 Jan 18;128(4):1644–53. doi: 10.1021/acs.jpcc.3c07477 (PMC10839897; doi:10.1021/acs.jpcc.3c07477)
Supplement: Supplementary file 1 — jp3c07477_si_001.pdf [file jp3c07477_si_001.pdf]

Supporting materials

Enhanced Biphasic Reactions in Amphiphilic Silica Mesopores

Guolin Zhao,[1,2] Yao Li,[1,2] Wen Zhen,[3] Jie Gao,[1] Yunjiao Gu,[1] Bing Hong,[1] Xia Han,[2] Shuangliang Zhao,[2,3*] Marc Pera-Titus[1,3*]

[1] Eco-Efficient Products and Processes Laboratory (E2P2L), UMI 3464 CNRS – Solvay, 3966 Jin Du Road, Xin Zhuang Ind. Zone, 201108 Shanghai, China

[2] State Key Laboratory of Chemical Engineering, East China University of Science and Technology, Shanghai 200237, China

[3] School of Chemistry and Chemical Engineering, Guangxi University, Nanning, 530004, China

[4] Cardiff Catalysis Institute, School of Chemistry, Cardiff University, Main Building, Park Place, Cardiff CF10 3AT, UK

Table of Contents

Figure S1. Coarse-grained models for (A) dodecanal (DA), (B) ethylene glycol (EG), (C1) super-SiNP based on 13 primary particles, (C2) primary particle and (C3) distribution of hydrophilic (HL) and hydrophobic (HB) groups on the primary particle.

Figure S2. Schematic structure of simulated super-SiNPs constituted by small primary particles, with variable pore volume. (A) 6NPs; (B) 7NPs; (C) 8NPs; (D) 9NPs; (E) 12NPs; (F) 13NPs.

Figure S3. Example of super-SiNP adsorbed at the DA/EG interface and calculation of the interfacial contact angle.

Figure S4. HR-TEM micrographs of the different Aerosil® particles used in this study.

Figure S5. Evolution of average size of droplets as a function of the average particle diameter of functionalized super-SiNPs. Emulsification conditions: DA/EG ratio = 2 : 1 (v/v), 50 mg NP, 50 °C, homogenization at 13,000 rpm for 5 min.

Figure S6. Evolution of the super-SiNP density at the DA/EG interface as a function of the pore volume of super-SiNPs. Emulsification conditions as in Figure S2.

Figure S7. Snapshots showing the position of super-SiNPs based on 6 (A1-F1) and 7 primary NPs (A2-F2) in the EG/DA system. Notation for A1-F2: DA (blue beads), EG (green beads), perimeter of super-SiNPs (yellow dotted line).

Figure S8. Snapshots showing the position of super-SiNPs based on 8 (A3-F3) and 9 primary NPs (A4-F4) in the EG/DA system. Notation for A3-F4: DA (blue beads), EG (green beads), perimeter of super-SiNPs (yellow dotted line).

Figure S9. Snapshots showing the position of super-SiNPs based on 12 (A5-F5) and 13 primary NPs (A6-F6) in the EG/DA system. Notation for A5-F6: DA (blue beads), EG (green beads), perimeter of super-SiNPs (yellow dotted line).

Figure S10. Evolution of DA/EG ratios in the pore volume of super-SiNPs measured from DPD simulations [$(DA/EG)_{DPD}$] against theoretical values estimated taking into account homogeneous pore filling by EG and DA from each phase for the particles adsorbed at the DA/EG interface [$(DA/EG)_{theo}$].

Figure S11. Pore slits with sizes in the range 2-5 nm functionalized with C_3SO_3H, C_8 and SiOH groups for GCMC simulations.

Figure S12. EG and DA concentration profiles in a 2-nm (A), 3-nm (B), 4-nm (C) and 5-nm (D) slits simulated by all-atom GCMC at variable EG:DA bulk volume ratios corresponding to different positions of adsorbed super-SiNPs at the DA/EG interface.

Table S1. Interaction parameters used in the DPD simulations.

Table S2. Physicochemical properties of emulsions stabilized by AX super-SiNPs

Table S3. Concentration of EG and DA in the pore volume of super-SiNPs computed by DPD.

Table S4. Average DA/EG ratios in the pore volume of super-SiNPs computed by DPD.

Table S5. Concentration of EG and DA in slits of different sizes computed by all-atom GCMC.

Table S6. Average DA/EG ratios in slits of different sizes computed by all-atom GCMC.

Table S7. DA-EG yield in the acetalization reaction of DA and EG in impregnated AX super-SiNPs using methods i-iii [a].

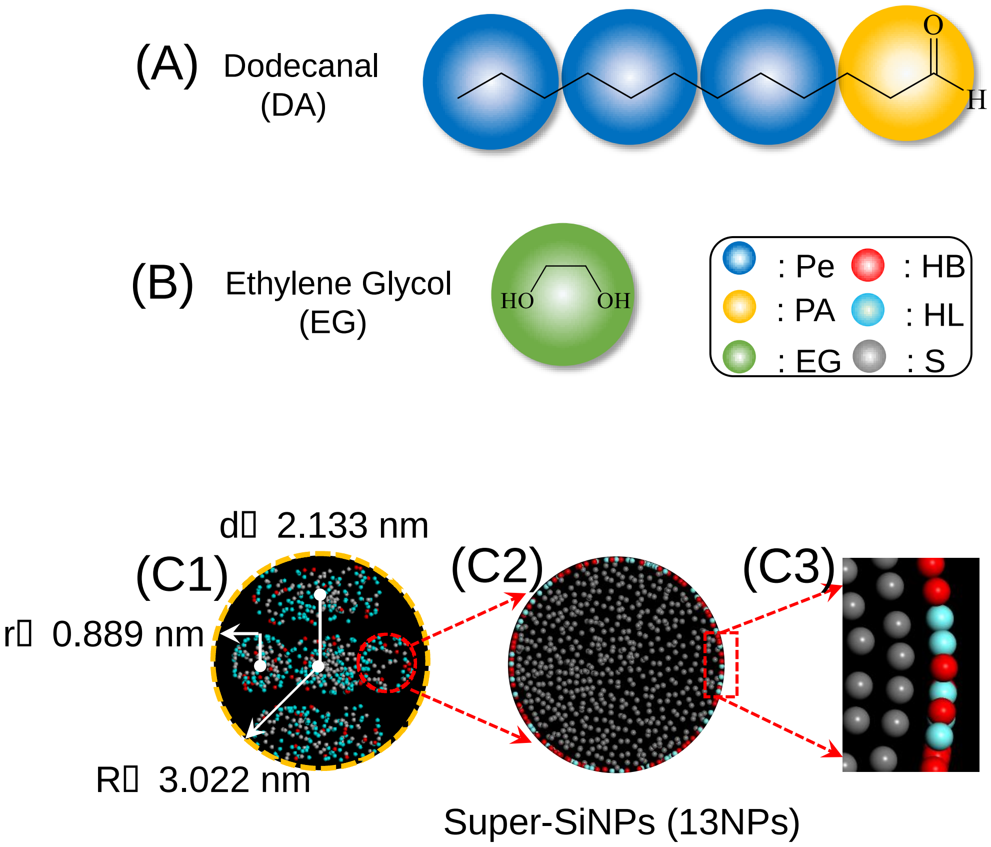

Figure S1. Coarse-grained models for (A) dodecanal (DA), (B) ethylene glycol (EG), (C1) super-SiNP based on 13 primary particles, (C2) primary particle and (C3) distribution of hydrophilic (HL) and hydrophobic (HB) groups on the primary particle.

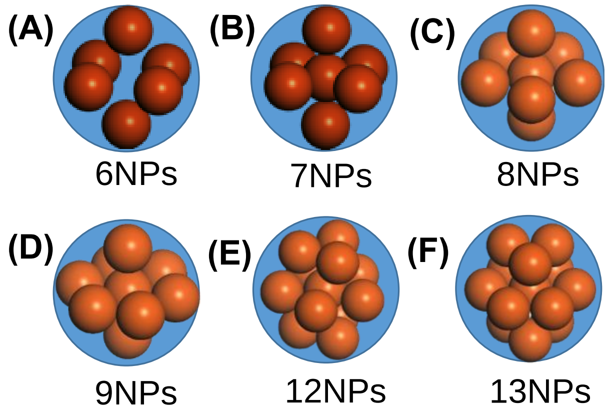

Number of primary NPs	6NPs	7NPs	8NPs	9NPs	12NPs	13NPs
V_p ($cm^3.g^{-1}$)	2.52	2.10	1.78	1.53	1.03	0.92

Figure S2. Schematic structure of simulated super-SiNPs constituted by small primary particles, with variable pore volume. (A) 6NPs; (B) 7NPs; (C) 8NPs; (D) 9NPs; (E) 12NPs; (F) 13NPs.

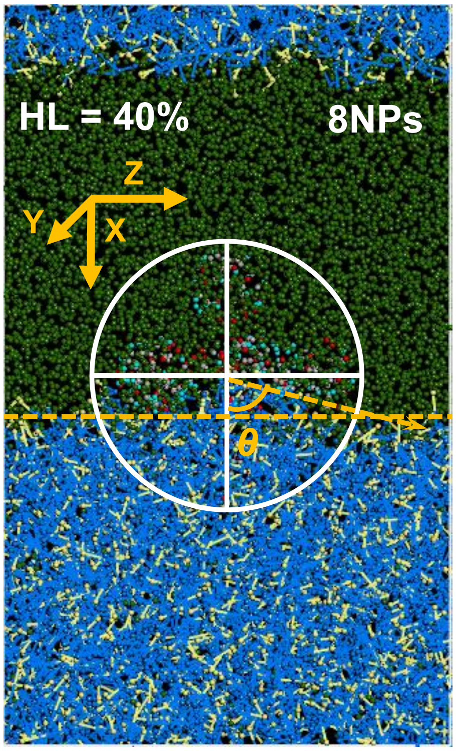

Figure S3. Example of super-SiNP adsorbed at the DA/EG interface and calculation of the interfacial contact angle.

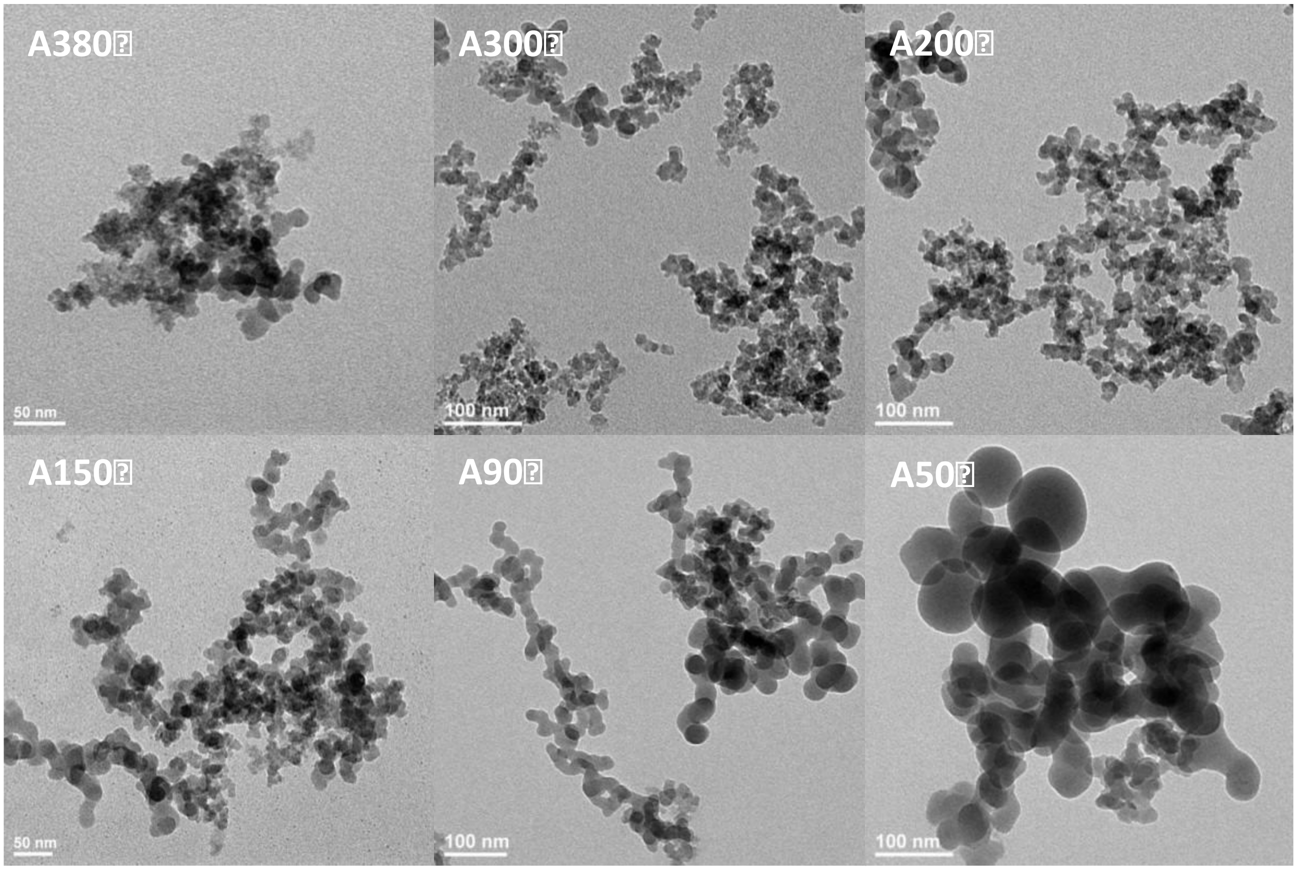

Figure S4. HR-TEM micrographs of the different Aerosil® particles used in this study.

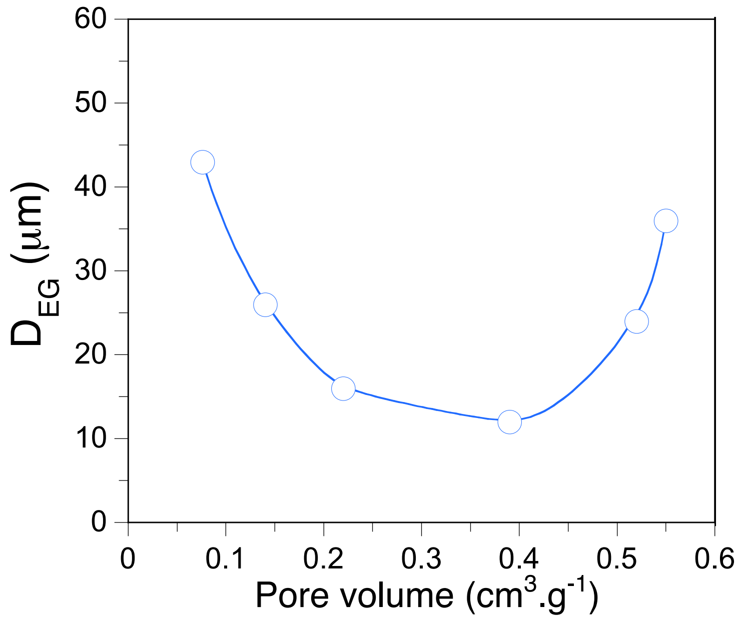

Figure S5. Evolution of average size of droplets as a function of the average particle diameter of functionalized super-SiNPs. Emulsification conditions: DA/EG ratio = 2 : 1 (v/v), 50 mg NP, 50 °C, homogenization at 13,000 rpm for 5 min.

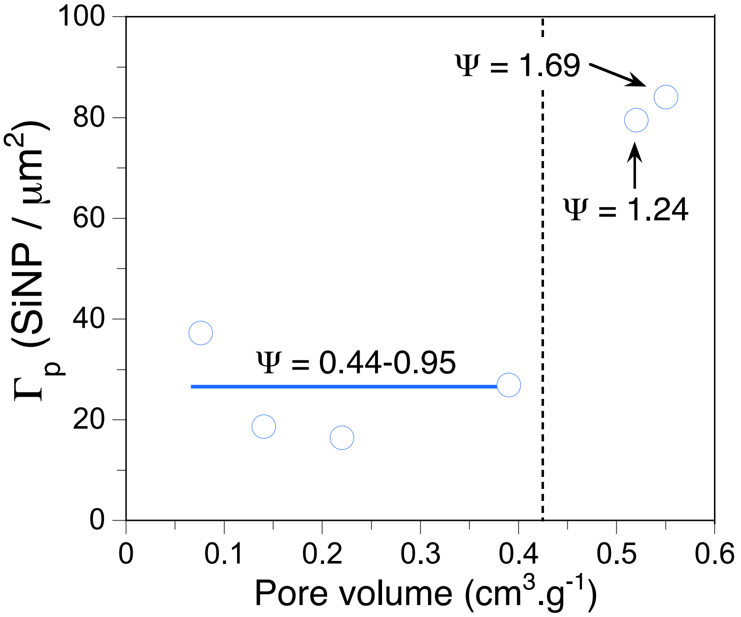

Figure S6. Evolution of the SiNP density at the DA/EG interface as a function of the pore volume of super-SiNPs. Emulsification conditions as in Figure S2.

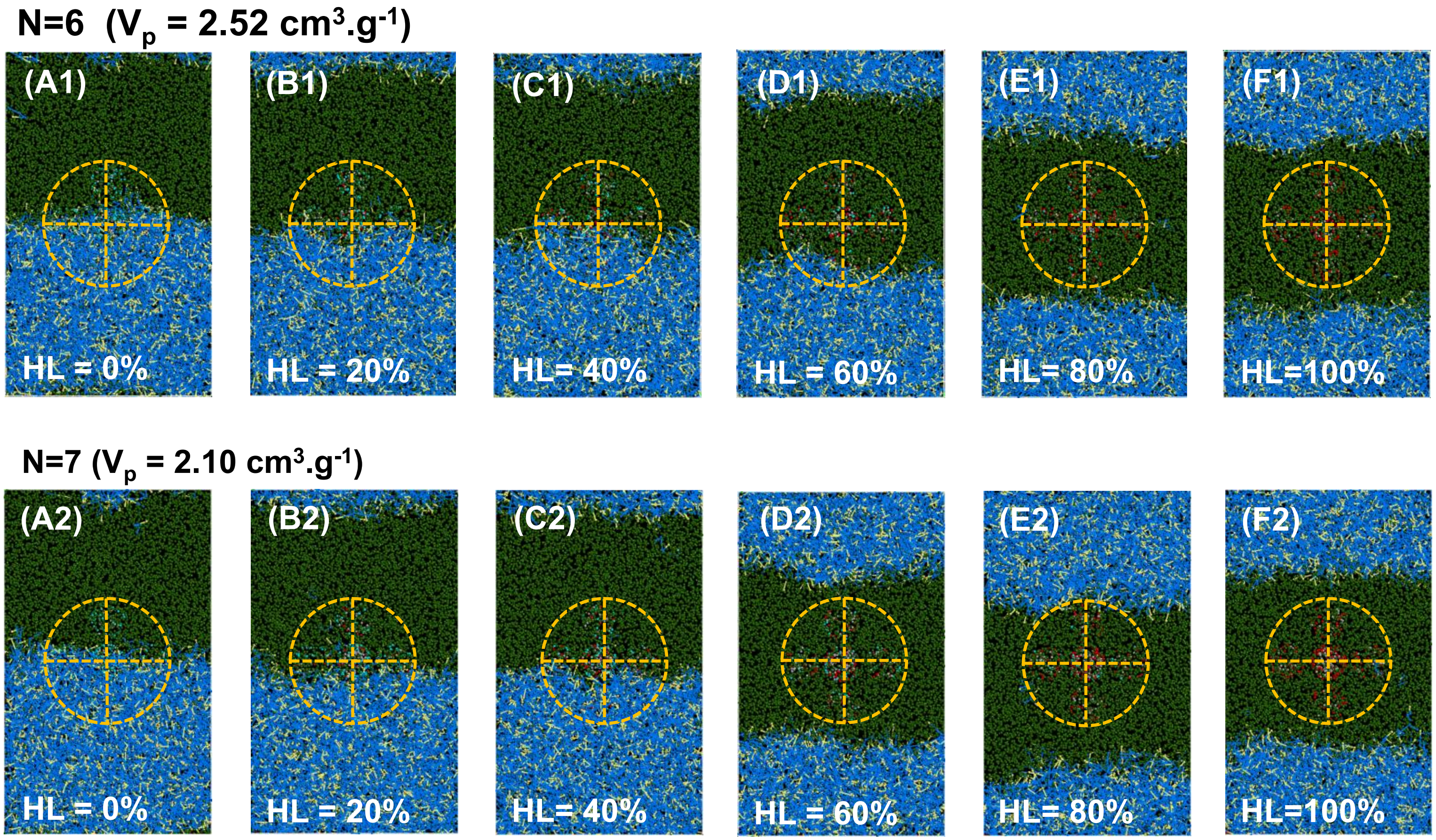

Figure S7. Snapshots showing the position of super-SiNPs based on 6 (A1-F1) and 7 primary NPs (A2-F2) in the EG/DA system. Notation for A1-F2: DA (blue beads), EG (green beads), perimeter of super-SiNPs (yellow dotted line).

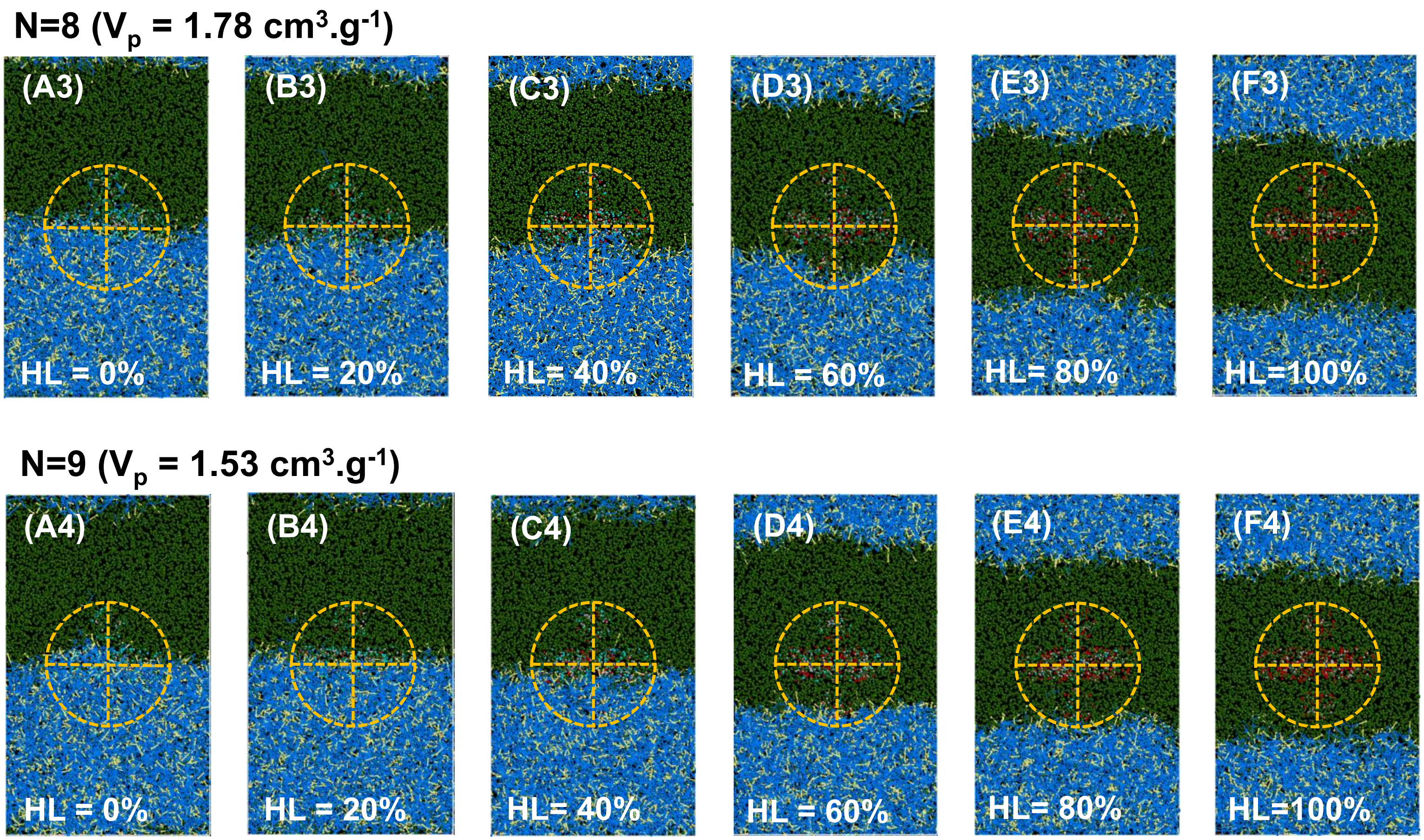

Figure S8. Snapshots showing the position of super-SiNPs based on 8 (A3-F3) and 9 primary NPs (A4-F4) in the EG/DA system. Notation for A3-F4: DA (blue beads), EG (green beads), perimeter of super-SiNPs (yellow dotted line).

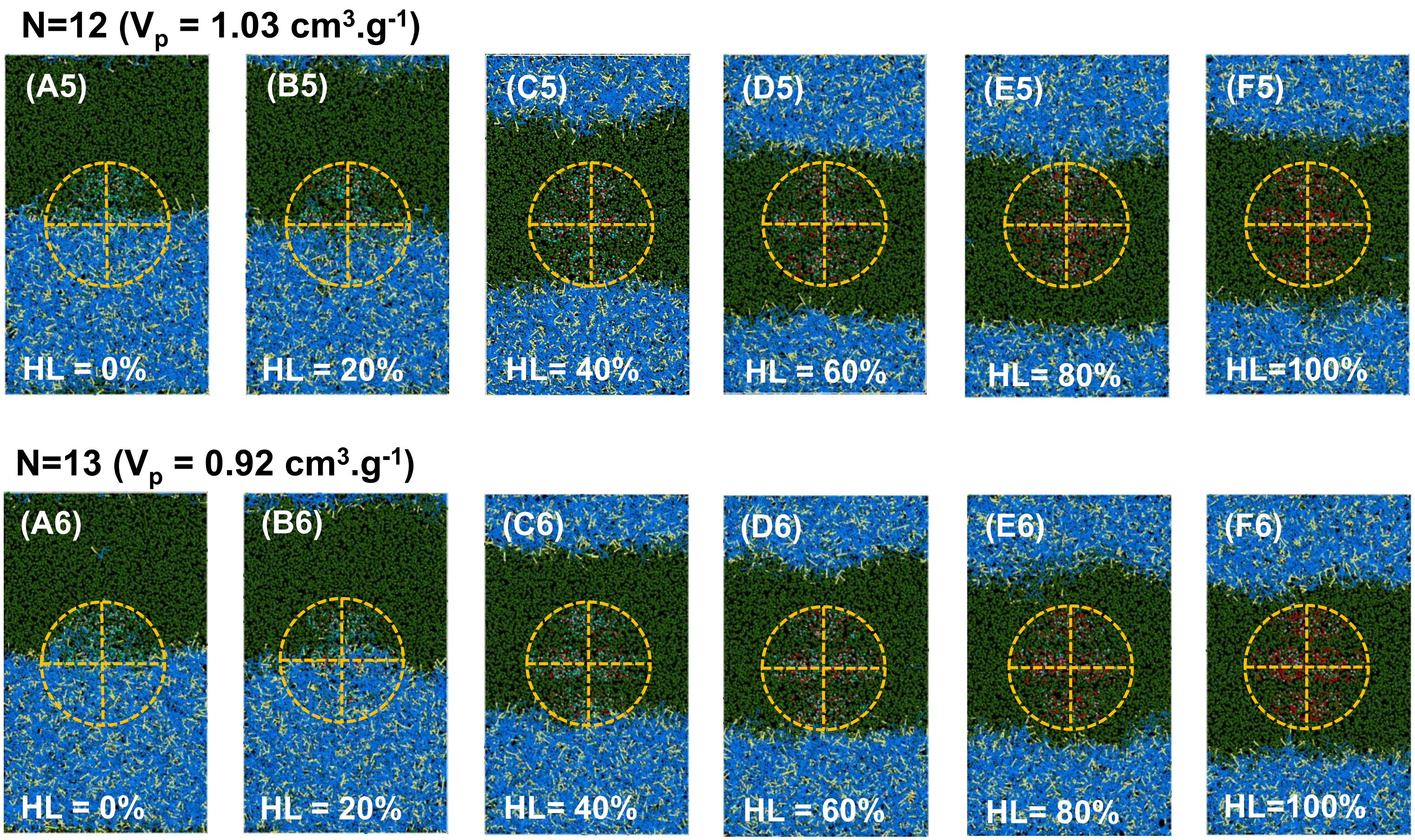

Figure S9. Snapshots showing the position of super-SiNPs based on 12 (A5-F5) and 13 primary NPs (A6-F6) in the EG/DA system. Notation for A5-F6: DA (blue beads), EG (green beads), perimeter of super-SiNPs (yellow dotted line).

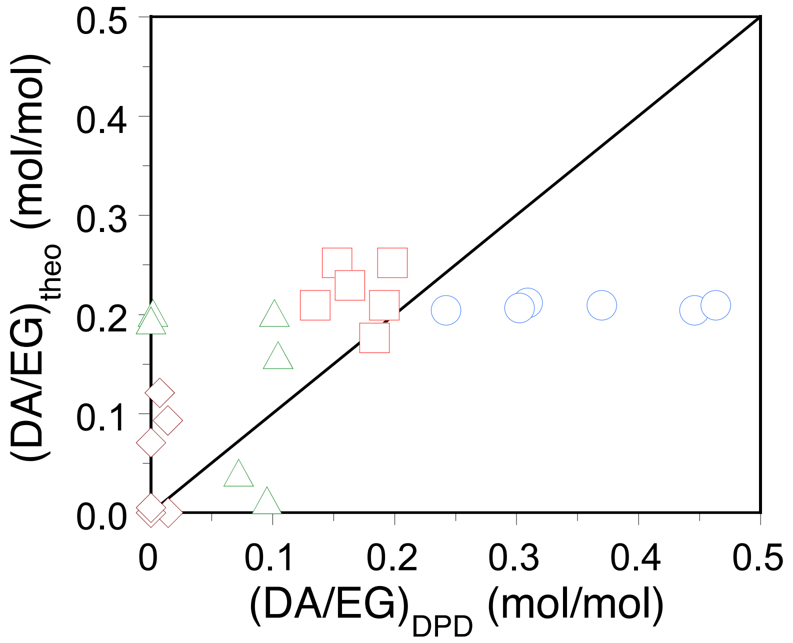

Figure S10. Evolution of DA/EG ratios in the pore volume of super-SiNPs measured from DPD simulations [$(DA/EG)_{DPD}$] against theoretical values estimated taking into account homogeneous pore filling by EG and DA from each phase for the particles adsorbed at the DA/EG interface [$(DA/EG)_{theo}$].

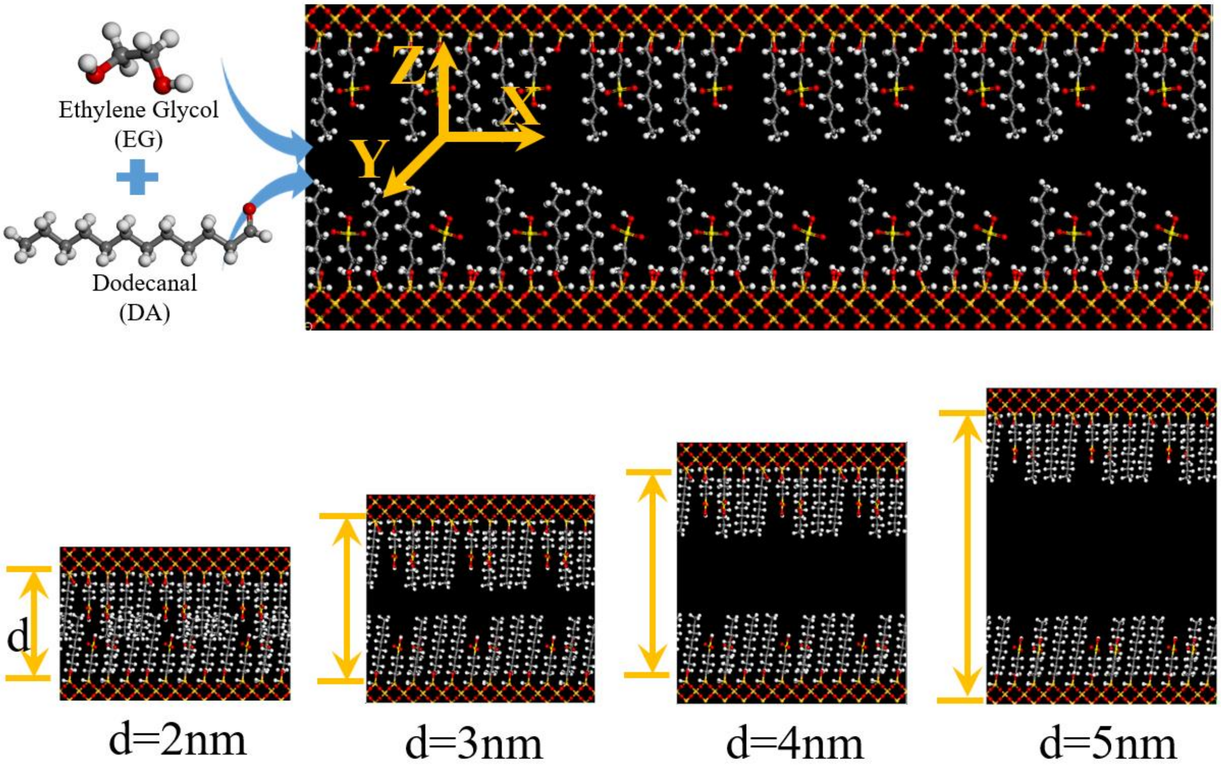

Figure S11. Pore slits with sizes in the range 2-5 nm functionalized with C_3SO_3H, C_8 and SiOH groups for GCMC simulations.

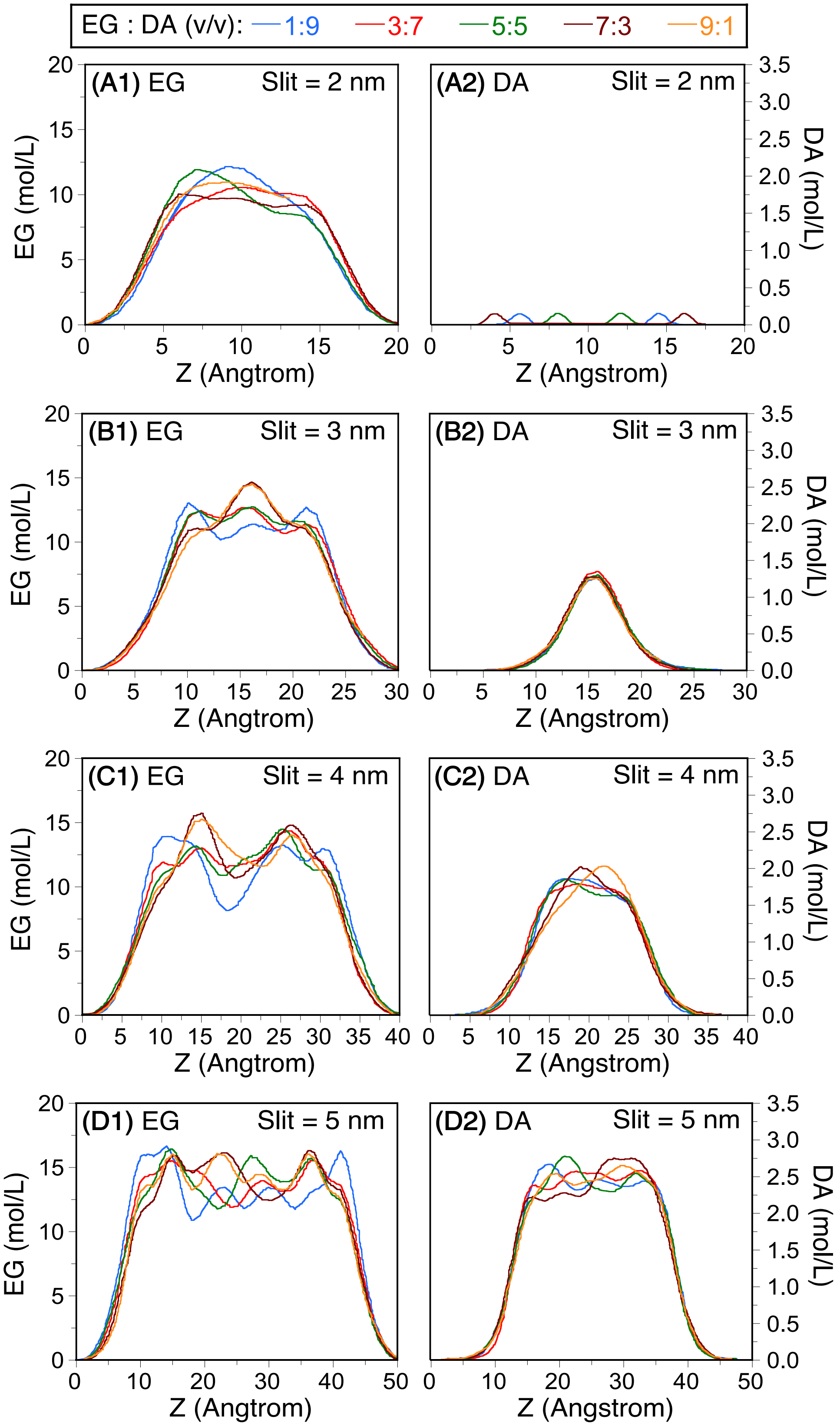

Figure S12. EG and DA concentration profiles in a 2-nm (A), 3-nm (B), 4-nm (C) and 5-nm (D) slits simulated by all-atom GCMC at variable EG:DA bulk volume ratios corresponding to different positions of adsorbed super-SiNPs at the DA/EG interface.

Table S1. Interaction parameters used in the DPD simulations

	**EG**	**Pe**	**PA**	**S**	**HB**	**HL**
**EG**	25.00	-	-	-	-	-
**Pe**	42.15	25.00	-	-	-	-
**PA**	28.09	30.68	25.00	-	-	-
**S**	30.50	67.08	41.83	25.00	-	-
**HB (=Pe)**	42.15	25.00	30.68	67.08	25.00	-
**HL (=H_2O/OH)**	25.21	46.20	29.93	28.54	46.20	25.00

Table S2. Physicochemical properties of emulsions stabilized by AX super-SiNPs

AX	D_{part} (nm)	Droplet size (μm)	$S_{E,int}$ (m^2)	Φ_{90} (-)	Γ_p (μm^{-2})
A380	160	36	0.28	1.69	84
A300	141	24	0.42	1.24	80
A200	153	12	0.84	0.50	27
A150	184	16	0.63	0.44	17
A90	199	26	0.39	0.58	19
A50	180	43	0.23	0.95	37

Table S3. Concentration of EG and DA in the pore volume of super-SiNPs computed by DPD

	HL%	6NPs	7NPs	8NPs	9NPs	12NPs	13NPs
EG (mol/L)	0.0	5.09	6.65	5.73	5.36	4.65	3.82
	0.2	8.64	7.27	7.72	6.93	7.65	6.22
	0.4	11.24	9.89	8.69	8.44	11.49	10.96
	0.6	14.09	13.84	12.27	12.45	11.95	11.30
	0.8	14.88	14.26	13.10	12.51	11.70	11.96
	1.0	14.58	14.12	13.15	12.57	12.23	12.26
DA (mol/L)	0.0	2.27	1.61	1.77	1.62	1.72	1.77
	0.2	1.32	1.44	1.26	1.27	1.03	1.19
	0.4	0.81	0.94	0.88	0.88	0.02	0.00
	0.6	0.20	0.00	0.17	0.09	0.00	0.00
	0.8	0.00	0.00	0.00	0.02	0.00	0.00
	1.0	0.00	0.00	0.00	0.00	0.00	0.00

Table S4. Average DA/EG ratios in the pore volume of super-SiNPs computed by DPD

HL%	6NPs	7NPs	8NPs	9NPs	12NPs	13NPs
0.0	0.45	0.24	0.31	0.30	0.37	0.46
0.2	0.15	0.20	0.16	0.18	0.14	0.19
0.4	0.07	0.09	0.10	0.10	0.00	0.00
0.6	0.01	0.00	0.01	0.01	0.00	0.00
0.8	0.00	0.00	0.00	0.00	0.00	0.00
1.0	0.00	0.00	0.00	0.00	0.00	0.00

Table S5. Concentration of EG and DA in slits of different sizes computed by all-atom GCMC

	EG:DA (bulk phase)	Molar ratio/slit			
		2nm	3nm	4nm	5nm
**EG (mol/L)**	1:9	6.36	7.11	8.49	10.61
	3:7	6.36	7.14	8.49	10.59
	5:5	6.36	7.11	8.51	10.66
	7:3	6.37	7.11	8.50	10.58
	9:1	6.37	7.10	8.50	10.57
**DA (mol/L)**	1:9	0.0226	0.287	0.699	1.29
	3:7	0.000	0.293	0.696	1.29
	5:5	0.0197	0.284	0.701	1.29
	7:3	0.0272	00.286	0.700	1.29
	9:1	0.000	0.285	0.700	1.29

Table S6. Average DA/EG ratios in slits of different sizes computed by all-atom GCMC

EG:DA (bulk phase)	Molar ratio/slit			
	2nm	3nm	4nm	5nm
1:9	0.00355	0.0403	0.0824	0.121
3:7	0.000	0.0410	0.0819	0.122
5:5	0.00310	0.0399	0.0824	0.121
7:3	0.00427	0.0402	0.0824	0.122
9:1	0.000	0.0401	0.0823	0.122

Table S7. DA-EG yield in the acetalization reaction of DA and EG in impregnated AX super-SiNPs using methods i-iii [a]

Super-SiNPs	Method i /%	Method ii /%	Method iii /%
A380*	93 (12)	37 (1.1)	92 (14)
A300	89	26	87
A200*	89 (0.71)	28 (0.73)	88 (0.77)
A150	89	21	87
A90	96	24	88
A50	100	19	99

[a] (i) First impregnation of EG, followed by DA; (ii) first impregnation of DA, followed by EG; (iii) co-impregnation of EG and DA.

* In parentheses, non-functionalized super-SiNPs.
